# Regulation of Rac1 and Reactive Oxygen Species Production in Response to Infection of Gastrointestinal Epithelia

**DOI:** 10.1371/journal.ppat.1005382

**Published:** 2016-01-13

**Authors:** Gerco den Hartog, Ranajoy Chattopadhyay, Amber Ablack, Emily H. Hall, Lindsay D. Butcher, Asima Bhattacharyya, Lars Eckmann, Paul R. Harris, Soumita Das, Peter B. Ernst, Sheila E. Crowe

**Affiliations:** 1 Department of Medicine, University of California, San Diego, La Jolla, California, United States of America; 2 Department of Pathology, University of California, San Diego, La Jolla, California, United States of America; 3 Department of Surgery, University of Virginia, Charlottesville, Virginia, United States of America; 4 National Institute of Science Education and Research (NISER), Bhubaneswar, India; 5 Division of Pediatrics, Unit of Gastroenterology and Nutrition, School of Medicine, Pontifical Catholic University, Santiago, Chile; University of Illinois, UNITED STATES

## Abstract

Generation of reactive oxygen species (ROS) during infection is an immediate host defense leading to microbial killing. APE1 is a multifunctional protein induced by ROS and after induction, protects against ROS-mediated DNA damage. Rac1 and NAPDH oxidase (Nox1) are important contributors of ROS generation following infection and associated with gastrointestinal epithelial injury. The purpose of this study was to determine if APE1 regulates the function of Rac1 and Nox1 during oxidative stress. Gastric or colonic epithelial cells (wild-type or with suppressed APE1) were infected with *Helicobacter pylori* or *Salmonella enterica* and assessed for Rac1 and NADPH oxidase-dependent superoxide production. Rac1 and APE1 interactions were measured by co-immunoprecipitation, confocal microscopy and proximity ligation assay (PLA) in cell lines or in biopsy specimens. Significantly greater levels of ROS were produced by APE1-deficient human gastric and colonic cell lines and primary gastric epithelial cells compared to control cells after infection with either gastric or enteric pathogens. *H*. *pylori* activated Rac1 and Nox1 in all cell types, but activation was higher in APE1 suppressed cells. APE1 overexpression decreased *H*. *pylori*-induced ROS generation, Rac1 activation, and Nox1 expression. We determined that the effects of APE1 were mediated through its N-terminal lysine residues interacting with Rac1, leading to inhibition of Nox1 expression and ROS generation. APE1 is a negative regulator of oxidative stress in the gastrointestinal epithelium during bacterial infection by modulating Rac1 and Nox1. Our results implicate APE1 in novel molecular interactions that regulate early stress responses elicited by microbial infections.

## Author Summary

*Helicobacter pylori* infection of the gastric mucosa is largely lifelong leading to continued stimulation of immune cells. This results in the generation of reactive oxygen species (ROS) which are produced to kill bacteria, but at the same time ROS regulate cellular events in the host. However, prolonged generation of ROS has been implicated in damage of DNA, which ultimately could lead to the development of cancer. We studied a molecule known as APE-1 in gastric and intestinal cells, which is activated upon encounter of ROS. Our results show that APE1 limits the production of ROS in cells that form the lining of the gastrointestinal tract. APE1 regulates ROS production by inhibiting activation of the molecule Rac1. Inhibition of ROS production by APE1 occurred after infection of gastric cells with *Helicobacter pylori* and after *Salmonella* infection of intestinal cells. These data demonstrate that APE1 inhibits production of ROS in cells that line the inside of the digestive tract.

## Introduction

The gastrointestinal epithelium serves as an initial interface between the host and luminal microbiota [1] and initiates innate immune responses to infection. Gastric and intestinal epithelial cells infected by microbial pathogens or commensal microbiota typically activate Rho GTPases leading, amongst other effects, to the production of reactive oxygen species (ROS) [2,3] that arise from the activation of the NADPH oxidase complex (Nox1) [4]. Nox1 family proteins are the catalytic, electron transporting subunits of Nox1 in non-phagocytic cells that produce superoxide [5,6]. While production of microbicidal levels of ROS in professional phagocytes via Nox2 is well-studied, information on ROS generation by gastric and intestinal epithelial cells in response to microbial signals via epithelial Nox1 is limited. The levels of ROS produced by epithelial cells are much lower than in phagocytes, and are more important in redox-sensitive signaling than direct antimicrobial killing. Nox1 is associated with the membrane-integrated protein p22^phox^, NOXA1 and NOXO1 to form superoxide [5]. Nox1 is expressed in gastric tissues [4] and is thought to play a role in ROS production in *H*. *pylori*-infected human gastric epithelial cells. While NADPH oxidase can be activated in epithelial cells throughout the gut, little is known about its responses to enteric infection.

*Helicobacter pylori* causes a lifelong infection that can lead to gastric and duodenal ulceration and gastric cancer, one of the major causes of cancer mortality worldwide [7,8,9]. Following *H*. *pylori* infection of guinea pigs [10], humans [11] and cultured gastric epithelial cells [12], an increase in oxidative stress occurs. *H*. *pylori* lipopolysaccharide (LPS) activates the small GTPase, Rac1, leading to Nox1 activation and production of superoxide [10,13,14,15]. Since *H*. *pylori* is a persistent infection, chronic ROS exposure eventually leads to oxidative DNA damage [4,16,17] and activation of signaling pathways implicated in the pathogenesis of cancer [18,19].

Accumulation of ROS increases APE1 activation [20] which in turn, mediates vital functions designed to protect the host [18]. APE1 is a multifunctional protein that is widely express in epithelial cells and that regulates multiple responses to bacterial infections, including chemokine production, apoptosis, cell proliferation and responses to hypoxia. The carboxy-terminus of APE1 is responsible for repairing DNA damage induced by ROS, while its N-terminal region regulates transcription [18]. Another distinct transcriptional regulatory role of APE1 is mediated by the N-terminal Lys6/Lys7 acetylation, which modulates certain promoter activities [21,22,23]. We have shown that APE1 is upregulated in gastric epithelial cells in the context of *H*. *pylori* infection [20] and contributes to the activation of AP-1 and NF-κB that regulate cell responses, including IL-8 production [24,25] and inhibition of cell death during *H*. *pylori* infection [26]. Interestingly, in a model of mouse hepatic ischemia/reperfusion, overexpression of APE1 resulted in suppression of reperfusion-stimulated oxidative stress [27]. While infection of gastric epithelial cells with *H*. *pylori* is a suitable model system to study the mechanisms of APE1-mediated regulation of ROS, *Salmonella enterica* serovar Typhimurium can be used as model to study the mechanisms of ROS production by intestinal epithelial cells (IEC). The pathogenicity of *Salmonella* is in part dependent on the presence of the *Salmonella* pathogenicity island 2 (SPI2) that interferes with ROS production by Nox2 in macrophages [28,29]. As many of the established infection-induced effects on gastrointestinal physiology are mediated by ROS-dependent mechanisms, we sought to compare the role of APE1 in ROS generation following infection with gastric or enteric pathogens.

In the current study, we provide evidence that *H*. *pylori*- and *Salmonella*-induced ROS is inhibited by APE1 in gastric and intestinal epithelial cells respectively. We also demonstrate that the Lys residues at the N-terminus of APE1 at positions 6 and 7, are required for Rac1 binding. This interaction inhibits Rac1 activation and Nox1 expression, decreasing ROS generation that results from infection. Together, our findings show a novel role of APE1 in regulating ROS levels in gastrointestinal epithelial cells following infection.

## Materials and Methods

### Bacterial strains, cell culture, transfection and plasmids

Empty retroQ vector (pSIREN), APE1 shRNA expressing (shRNA) cells, or non-transfected AGS (AGS) cells obtained from American Type Culture Collection were harvested and cultured in Ham’s F/12 medium (Hyclone) supplemented with 10% heat-inactivated FBS (Hyclone) [21]. NCI-N87 cells obtained from ATCC were maintained in RPMI supplemented with 10% FBS. T84 and HT-29 cells (a kind gift from Dr. K. Barrett, University of California San Diego) were maintained respectively in L-Glutamine containing F12/DMEM supplemented with 5% FBS and in McCoy’s 5A medium supplemented with 10% FBS. *H*. *pylori* 26695, a *cag* PAI^+^ strain (ATCC) and its isogenic mutants, *cag* PAI^−^ strain 8–1 and VacA (kind gift from Dr R.M. Peek, VanderBilt University, Tennessee, USA [30]), were maintained as previously described [21]; a MOI of 100 was used for all the experiments in this study as this was the highest dose with minimal necrotic cell death [26]. Previously, we reported that infection of gastric epithelial cells with *H*. *pylori* longer than 6h cause cell death and therefore, longer infection times do not result in reliable ROS data [26].

Gastric antrum-derived primary epithelial cells were isolated and maintained in culture according to the procedures developed by Dr. Stappenbeck [31]. Briefly, biopsy samples were obtained from consenting adult patients undergoing esophagogastroduodenoscopy (IRB UCSD HRPP 150476) were minced in small pieces and treated with collagenase at 37°C for approximately 1h. Then cells were washed and filtered. Cultures were maintained in matrigel and medium containing Wnt3a, R-spondin and Noggin, which was refreshed or passaged every other day. For luminol experiments, wells were coated with 1/30 matrigel for 30 min, which was removed immediately before cells were added and for imaging, glass slides were coated with 10 μg/cm^2^ with Collagen IV for 1.5h at 37°C, and washed with warm PBS prior to the addition of cells.

*Salmonella enterica* serovar Typhimurium strain SL1344 and a ΔSPI2 mutant (kind gifts from Drs. Olivia Steel Mortimer NIAID, Rocky Mountain Laboratory, Montana, USA and Brett Finlay, University of British Columbia, Canada), were used at MOI 30 in cultures of T84 cells and HT-29 cells. *Salmonella* cultures were grown as described previously [32]. Briefly, a single colony was inoculated into LB broth and grown for 8h under aerobic conditions and then under oxygen-limiting conditions overnight.

Wild type APE1, an N-terminal acetylation mutant of APE1 (N-K6R/K7R), and a C-terminal DNA repair mutant of APE1 (C-H309N) constructs were used as previously reported [26]. Active Rac1 V12 and dominant negative Rac1 N17 plasmids were kind gifts from Dr. Jim Casanova University of Virginia, Charlottesville, Virginia, USA. All epithelial cells were seeded in six-well plates 18–24h before transfection. For overexpression studies, cells were transfected using 2 μg of plasmid DNA with Lipofectamine 2000 reagent (Invitrogen) as per the manufacturer’s protocol. In keeping with the manufacturer’s recommendation cells were used for infected experiments 40h post-transfection.

### Nox1 suppression

*Nox1* expression was suppressed with human NOX1 siRNA ON-TARGETplus SMARTpool (Dharmacon RNAi technologies, L-010193-00-0005). AGS cells in 6 well plates were transfected using Lipofectamine RNAiMAX transfection reagent according to the protocol and luminol oxidation was measured after 48h.

### Antibodies and reagents

Antibodies used include the following: anti-APE1, mouse monoclonal anti-Nox1 (Novus Biologicals), rabbit polyclonal anti-APE1, mouse monoclonal anti-Rac1 clone 28A (Millipore) followed by incubation with anti-rabbit or anti-mouse HRP-conjugated IgG (Cell Signaling Technology). NADPH oxidase inhibitor diphenyleneiodonium (DPI) and Rac inhibitor NSC23766 were purchased from Calbiochem.

### Measurement of ROS

ROS in AGS and T84 cells were measured according to the protocol described in Lumimax Superoxide Anion Detection Kit (Stratagene). See S1 Supplementary Materials and Methods for details. Measurements of ROS in NCI-N87, HT-29 and primary gastric epithelial cells were performed using 1 mM luminol (Sigma A8511, without additional enhancers) dissolved in borax buffer (pH 9) and the Spectramax L (Molecular Devices) reader for detection. For microscopic detection of ROS, cells were loaded with 5 μM CM-H_2_DCFDA (Invitrogen) for 30 min in an incubator (5% CO_2_ 37°C). Following loading with CM-H_2_DCFDA cells were washed and infected.

### Western blotting and immunoprecipitation

Protein expression of APE1, Rac1 and Nox1 was assessed by western blot. Co-immunoprecipitation experiments were performed using anti-FLAG M2 agarose beads (Sigma) to analyze components that bind to FLAG-APE1 or FLAG-Rac1. See S1 Supplementary Materials and Methods for details. Densitometry was performed using ImageJ (National Institutes of Health). The levels of the protein of interest were corrected for the levels of the loading control (e.g. α-Tubulin).

### Measurement of Rac1 activation

Rac1 activity was measured as described previously [32] (see S1 Supplementary Materials and Methods). Densitometry was performed using ImageJ. The levels of active Rac1 were normalized for levels of total Rac1.

### Real time RT-PCR from gastric biopsies

cDNAs obtained from antral gastric biopsies of *H*. *pylori* infected and uninfected patients were kindly provided by Richard Peek, Vanderbilt University (Tennessee, USA). Additionally, antral gastric mucosa biopsy specimens were collected from *H*. *pylori*-infected and uninfected individuals during diagnostic esophagogastroduodenoscopy following a University of Virginia Human Investigation Committee (HIC) (IRB number 9686) approved protocol into HBSS with 5% FBS [21]. All patient samples were de-identified apart from being known to be *H*. *pylori* infected or uninfected. The samples were analyzed at the University of Virginia, Virginia USA. See S1 Supplementary Materials and Methods for details.

### Proximity ligation assay (PLA) by confocal microscopy

APE1-Rac1 interactions were detected with Duolink PLA Kit (Olink Bioscience, Uppsala, Sweden: PLA probe anti-rabbit plus; PLA probe anti-mouse minus; Detection Kit orange) according to the manufacturer's protocol. See S1 Supplementary Materials and Methods for details. Biopsy specimens for immunohistochemistry were obtained with Institutional Review Board approval of the Pontifical Catholic University, Santiago, Chile (IRB number 12–236) from adult subjects with abdominal symptoms in Santiago, Chile. Samples were collected and *H*. *pylori* status was determined by rapid urease test and microscopic evaluation, and a study subject was judged colonized with *H*. *pylori* if one or both tests were positive for the bacteria. In collaboration with Dr. Harris, these snap frozen samples were shipped to UCSD where PLA was performed. Quantification of co-localization was performed using the colocalization plugin (JACoP) for ImageJ which calculates Pearson’s coefficient. ImageJ was used to quantify the amount of PLA signal, which was corrected for the number of cells present in each field of view.

### Statistical analysis

Results are expressed as mean ± SEM. Statistical differences were calculated using ANOVA for multiple comparisons and Bonferroni post-hoc testing in Graphpad Prism. Levels of significance are indicated as follows: * p<0.05, ** p<0.01 and *** p< 0.001.

### Accession numbers

Proteins studied in this manuscript are given below with a reference to the SwissProt database:

APE1 (gene name APEX1), P27695

Rac1 (gene name RAC1), P63000

Nox1 (gene name NOX1), Q9Y5S8

## Results

### H. pylori-induced ROS generation is Rac1 dependent

**Fig 1:**
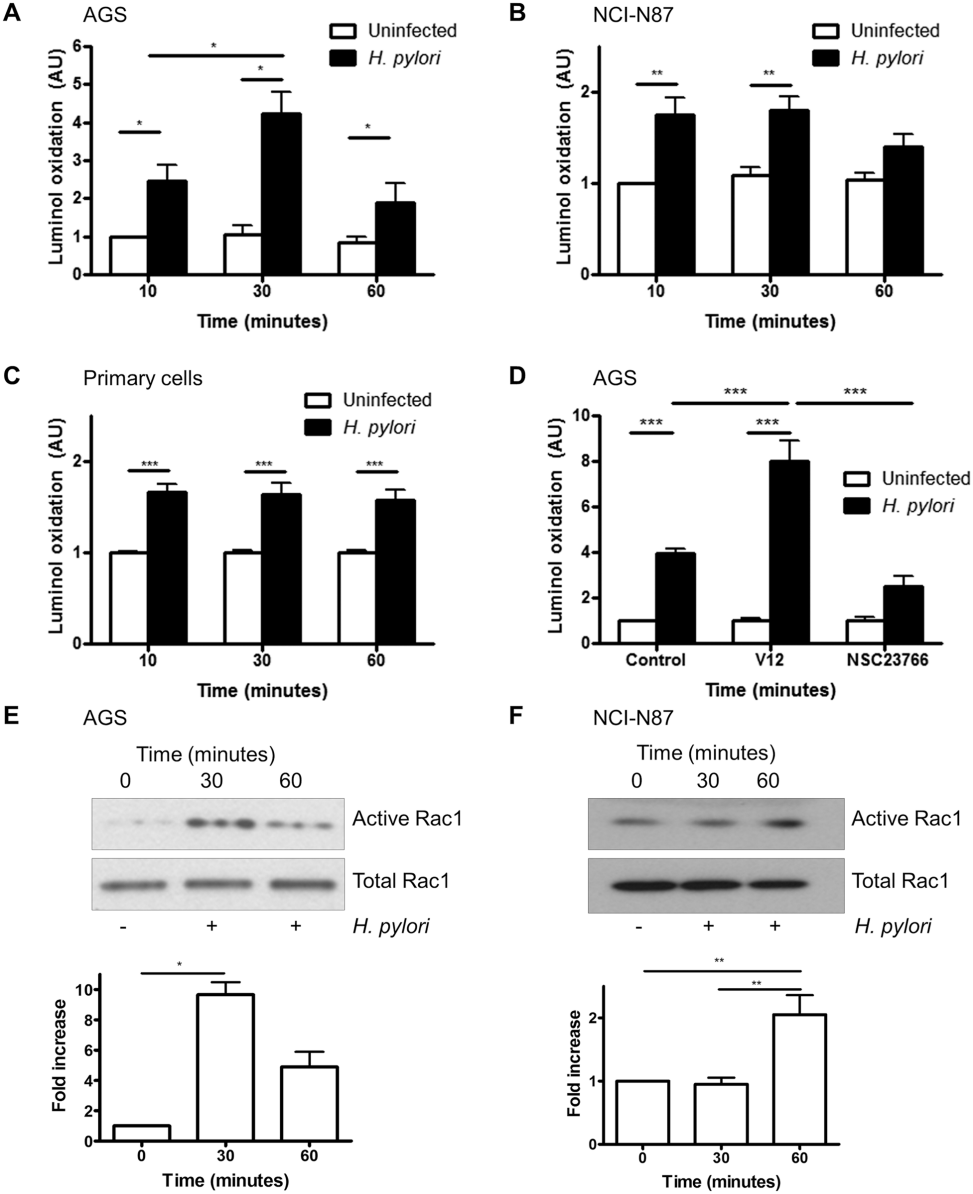
H. pylori-induced ROS generation is mediated by Rac1. (A-C) ROS generation was measured by luminol oxidation in AGS cells (A), NCI-N87 cells (B) and primary gastric epithelial cells (C) after infection with H. pylori 26695 for 10, 30 or 60 min or left uninfected. (D) H. pylori-induced ROS generation (after 30 min) was measured in AGS cells after transfection with empty vector (pcDNA), constitutively active Rac1 (V12) or after treatment with Rac1 inhibitor (NSC23766). For A-C fold change of luminol oxidation compared to uninfected cells are shown as mean values (± SEM) of three independent experiments. (E-F) Rac1 activation was measured by a GTP pull down assay in AGS (E) and NCI-N87 (F) cells following infection with H. pylori at the indicated times. A representative western blot was selected from three independent experiments and densitometry results from multiple experiments are shown. Levels of significance are indicated as follows: * p<0.05, ** p<0.01 and *** p< 0.001.

We observed a rapid increase in superoxide production in the human gastric adenocarcinoma-derived cell line AGS following infection with *H*. *pylori* (Fig 1A). To determine whether the production of ROS observed was not unique to AGS cells additional experiments were performed in an alternative cancer-derived cell line NCI-N87 and non-transformed antral-derived primary epithelial cells. Induction of ROS production was also observed in NCI-N87 cells and primary human gastric epithelial cells isolated from the antrum (Fig 1B and 1C respectively) following infection with wild type *H*. *pylori* strain 26695 although the kinetics where somewhat different from AGS cells. Superoxide generation by luminol oxidation was independent of the *vacA* and *cagA* pathogenicity island (PAI) status of *H*. *pylori* since no significant differences were seen when AGS cells were infected with wild type *H*. *pylori* or the *vacA* or *cagA* PAI mutant strain, 8–1 (S1 Fig). Prolonged infection studies showed that ROS is generated early following infection and not observed at 4h of infection or later (S2 Fig).

It is known that *H*. *pylori* activates Rac1, and another report shows that Rac1 activation initiates ROS production in guinea pig gastric cells [13,33]. To determine if Rac1 regulates *H*. *pylori*-mediated ROS generation in human gastric epithelial cells, a constitutively active Rac1 plasmid (V12) was overexpressed in AGS cells or cells were treated with the Rac1-specific inhibitor NSC23766, before *H*. *pylori* infection. Overexpression of active Rac1 resulted in increased ROS generation, while the Rac1 inhibitor reduced ROS generation compared to vector-transfected cells (Fig 1D). To confirm Rac1 activation during *H*. *pylori* infection, active Rac1 was assessed using a pulldown assay. As shown in Fig 1E and 1F, *H*. *pylori* 26695 infection increased Rac1 activation in AGS cells in 30 min and in NCI-N87 cells at 60 min after infection. Since there was no difference in ROS generation or Rac1 activation by *H*. *pylori* strains 26695 and 8–1 (S3 Fig), subsequent experiments were performed with *H*. *pylori* 26695 only. Although activation of Rac1 by *H*. *pylori* has been previously reported, here we expand this finding by showing that Rac1 is involved in the production of ROS by gastric epithelial cells following infection with *H*. *pylori*.

### H. pylori-induced ROS is mediated through NADPH oxidase

**Fig 2:**
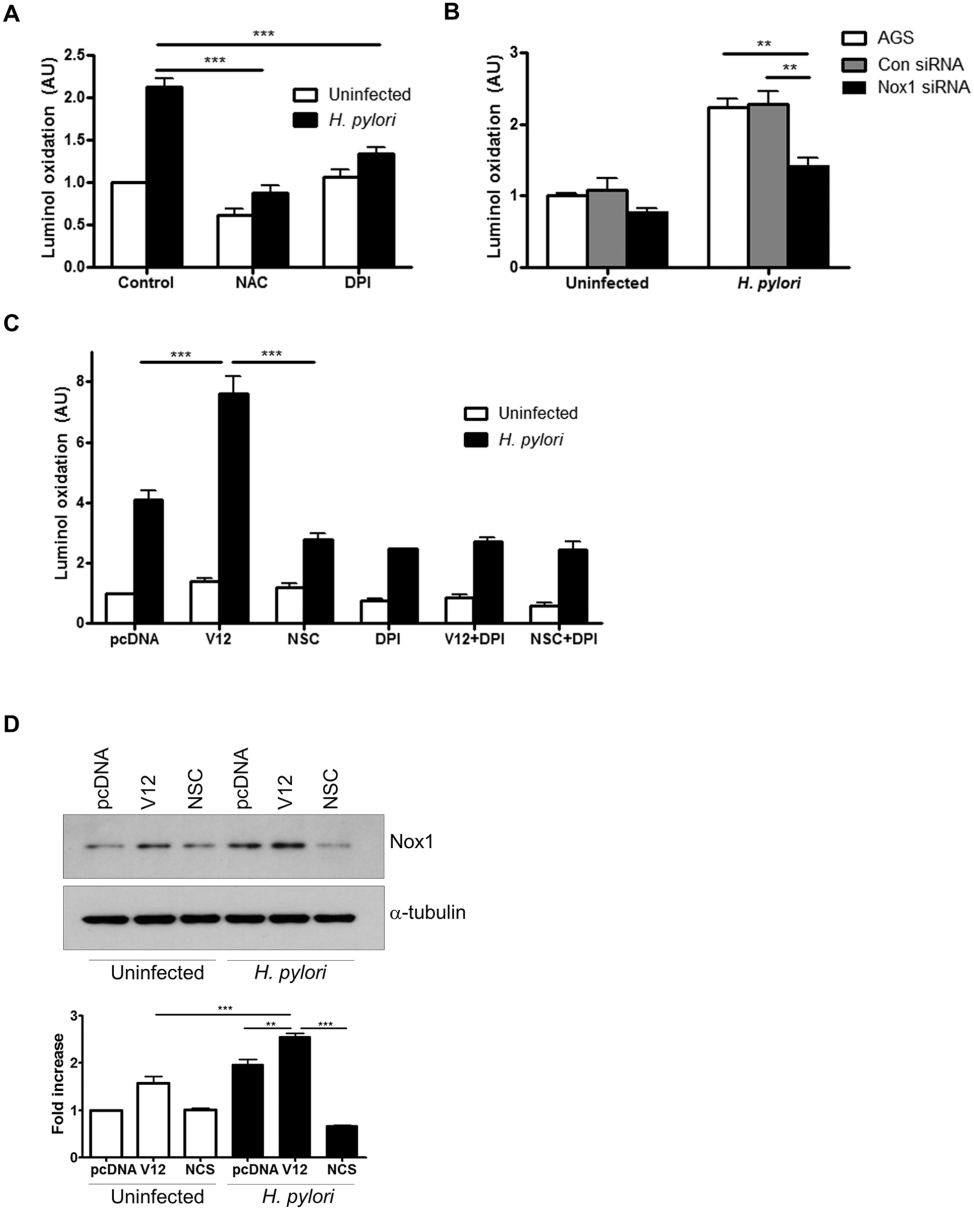
H. pylori-induced ROS generation is regulated by NADPH oxidase. ROS generation was measured in (A) AGS cells infected with H. pylori for 30 min or left uninfected in the presence of 10 mM NAC or 10 μM DPI, (B) in Nox1 downregulated AGS cells following infection with H. pylori for 30 min or left uninfected and (C) in AGS cells either transfected with pcDNA or Rac1 V12 following infection with H. pylori for 30 min. In other experimental conditions, AGS cells were treated with NSC23766 (NSC), or DPI or both before infection with H. pylori for 30 min. For A-C fold change of luminol oxidation compared to uninfected cells are shown as mean values (± SEM) of three independent experiments. (D) Nox1 expression was measured in uninfected and H. pylori infected AGS cells transfected with either vector (pcDNA) or Rac1 V12 or were pre-treated with NSC23766 (NSC). The representative western blot with Nox1 expression was selected from three independent experiments and densitometry results are shown. Levels of significance are indicated as follows: ** p<0.01 and *** p< 0.001.

After establishing that *H*. *pylori* induce ROS production by gastric epithelial cells through activation of Rac1, we investigated whether the ROS were generated by Nox1 as a major NADPH oxidase expressed in gastric epithelial cells [4]. Our results demonstrate that AGS cells infected in the presence of the general ROS inhibitor N-acetyl-L-cysteine (NAC), showed significant inhibition of superoxide production (Fig 2A). Also, infection in the presence of the NADPH oxidase inhibitor diphenyleneiodonium (DPI) resulted in inhibition of superoxide production, suggesting that NADPH oxidase is involved in *H*. *pylori*-induced ROS generation. As DPI is not a specific inhibitor of Nox1, we used siRNA-mediated suppression of Nox1 to show a comparable decrease in luminol oxidation following infection with *H*. *pylori* ROS (Fig 2B). To evaluate the relative contributions of Nox1 and Rac1 in *H*. *pylori*-induced ROS generation, luminol oxidation was measured in AGS cells in the presence of DPI, the Rac1 inhibitor NSC23766 or overexpression of active Rac1. Comparable inhibition of ROS generation was observed when NSC23766 or DPI was used alone or in combination, indicating that Rac1 and NADPH oxidase share the same pathway to generate ROS. The increase in ROS in the presence of V12 was abrogated by DPI suggesting that Rac1 activation alone is not sufficient to generate ROS when NADPH oxidase activity is inhibited (Fig 2C). In a parallel experiment, Nox1 protein expression was increased in AGS cells within 1h of *H*. *pylori* infection. This induction was further enhanced in the presence of active Rac1 but decreased in the presence of the Rac1 inhibitor NSC23766 (Fig 2D). Together, our data demonstrate that NOX1 is the major source of ROS in gastric epithelial cells infected with *H*. *pylori*.

### APE1 inhibits H. pylori-induced ROS generation

**Fig 3:**
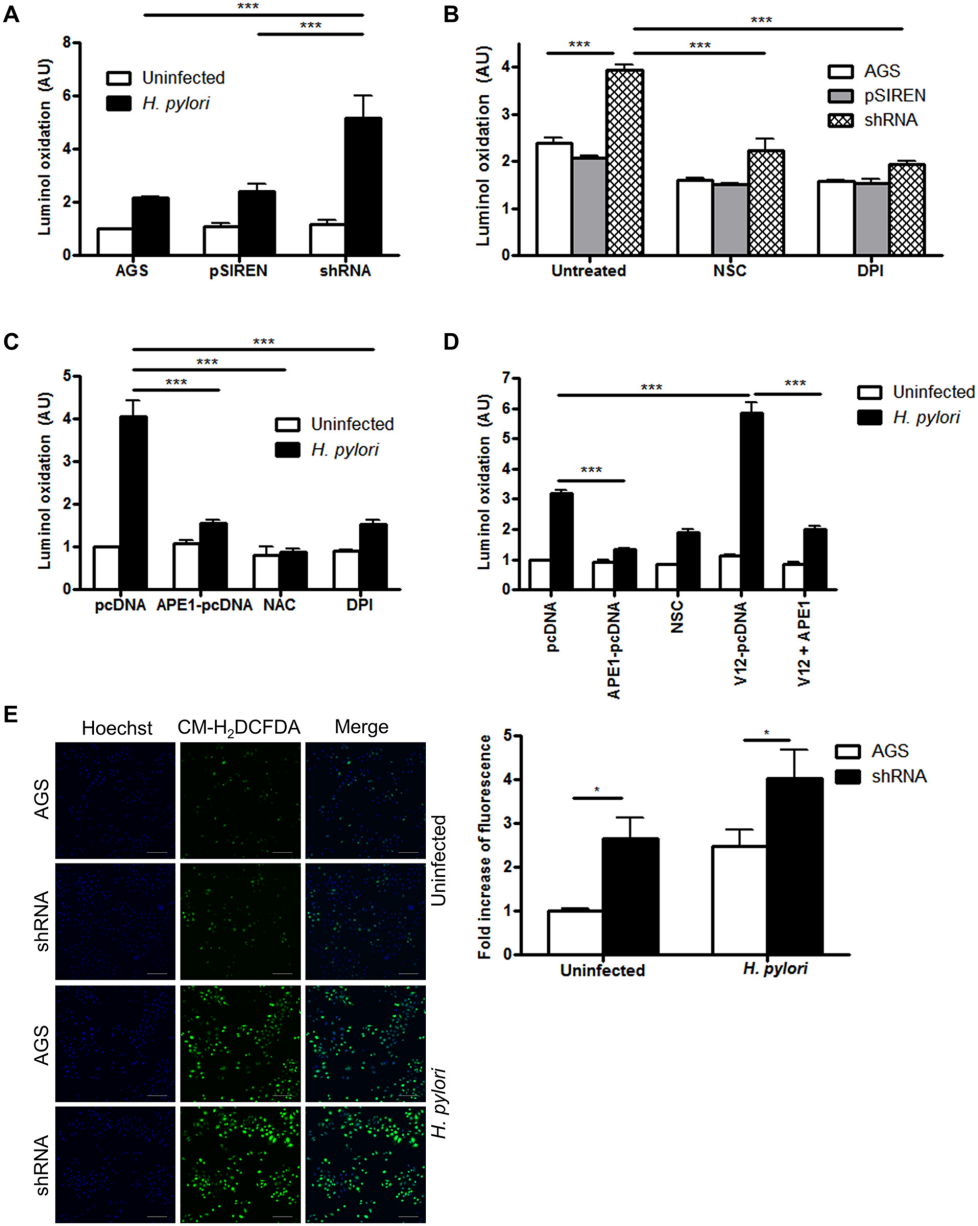
APE1 inhibits H. pylori-induced ROS generation. ROS generation was measured by luminol oxidation (A) in WT AGS, control shRNA (pSIREN) or APE1 shRNA (shRNA) cells after infection with H. pylori for 30 min or left uninfected, (B) in infected cells that were either left untreated or treated with NSC23766 or DPI, (C) in shRNA cells either transfected with empty vector (pcDNA) or APE1; or treated with NAC or DPI before infection with H. pylori for 30 min or left uninfected and (D) in shRNA cells that were either transfected with pcDNA, APE1, V12 or both APE1 and V12 or treated with NSC23766 before infection with H. pylori or left uninfected. For A-D fold change of luminol oxidation compared to uninfected cells are shown as mean values (± SEM) of three independent experiments. (E) ROS generation was measured by confocal microscopy in AGS and shRNA cells after infection with H. pylori for 30 min or left uninfected. After infection ROS was detected using CM-H_2_DCFDA and nuclei were stained with DAPI. Scale bar indicates 100 μm. The bar graph at the bottom shows quantification of multiple images from three independent experiments. Levels of significance are indicated as follows: * p<0.05, ** p<0.01 and *** p< 0.001.

It is known that ROS induces APE1, but whether APE1 modulates ROS generation has not been previously examined. As illustrated in Fig 3A, luminol oxidation was increased in APE1 suppressed cells indicating regulation of ROS by APE1. Corroborating the findings with luminol, immunofluorescence with CM-H_2_DCFDA demonstrated increased ROS generation in APE1 suppressed cells following infection (Fig 3E). The additional increase of ROS in APE1 suppressed cells was absent in the presence of NSC23766 or DPI suggesting that both Rac1 and NADPH oxidase act downstream of APE1 in the pathway of ROS generation (Fig 3B). Furthermore, overexpression of exogenous APE1 in cells with suppressed endogenous APE1 significantly reduced *H*. *pylori*-induced ROS generation (Fig 3C). APE1 overexpression was also sufficient to inhibit ROS generation in the presence of V12 overexpression implicating APE1 as a major regulator of Rac1-mediated oxidative stress (Fig 3D).

### APE1 interacts with Rac1 and inhibits Rac1 activation

**Fig 4:**
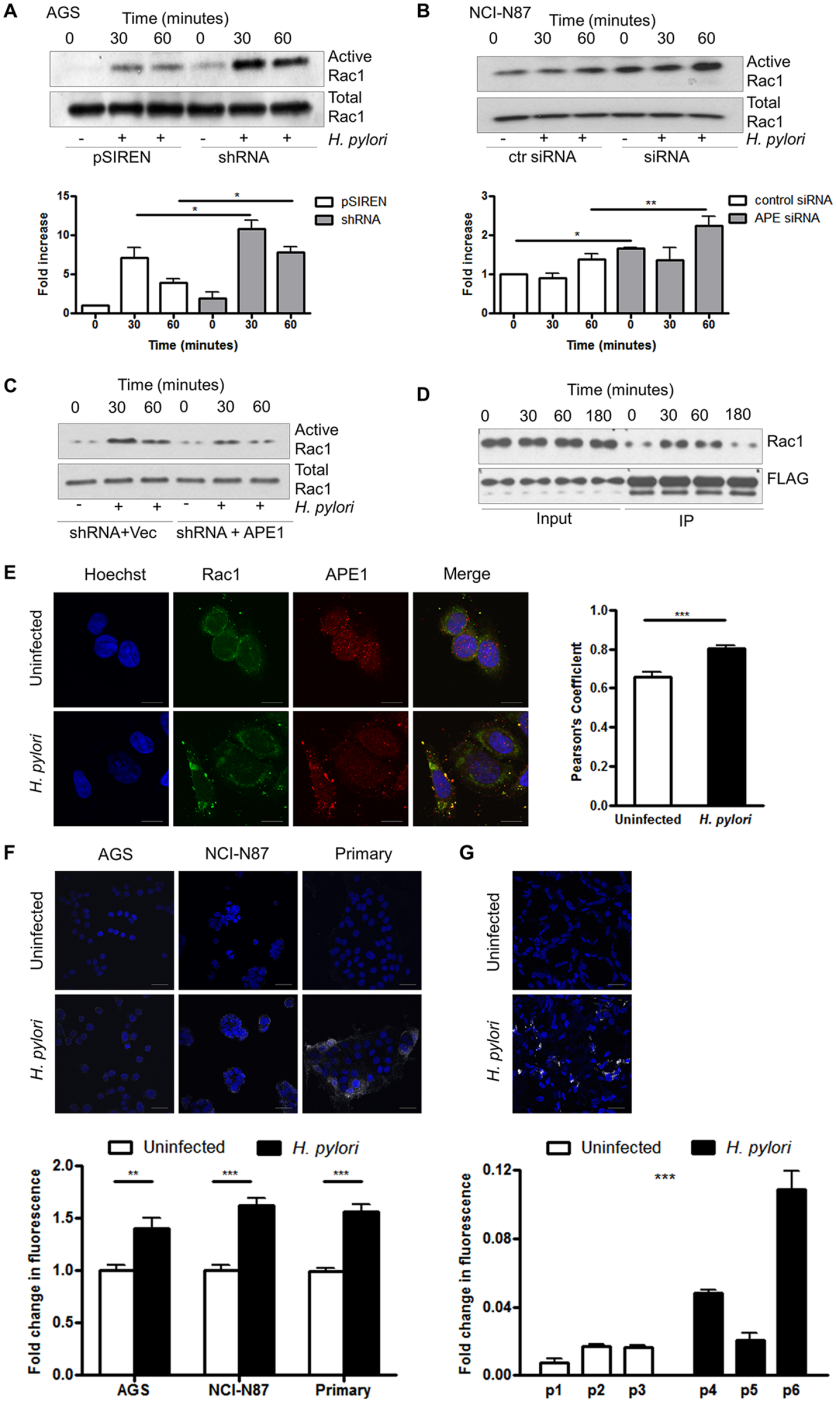
APE1 inhibits Rac1 activity and interacts with Rac1. (A-B) pSIREN and shRNA cells were infected with H. pylori for 30 min or 1h or left uninfected and Rac1 activity was measured by detecting levels of GTP-bound Rac1 in AGS (A) and NCI-N87 (B) cells that were transfected with control siRNA or siRNA against APE1. Bar graphs in A and B show densitometry results. (C) shRNA cells either transfected with vector control or APE1 were infected with H. pylori and Rac1 activity was measured as in A. (D) AGS cells were transfected with either vector control or FLAG tagged APE1 and infected with H. pylori for 30, 60 or 180 min or left uninfected. FLAG was immunoprecipitated levels of bound Rac1 determined. (A-D) Representative western blots from three independent experiments are shown. (E) Representative confocal microscope images demonstrating co-localization of Rac1 and APE1. AGS cells were either left untreated (top panels) or treated with H. pylori for 1h (bottom panels). Cells were stained for nuclei (Hoechst), APE1 or Rac1. Yellow pixels demonstrated the co-localization of APE1 with Rac1 and scale bars indicate 10 μm. Results of the quantification by calculating Pearson’s coefficient are shown in the bar graph (F) APE1-Rac1 proximity was visualized using a proximity ligation assay (PLA). Each white spot represents APE1-Rac1 interaction in uninfected (top) and H. pylori infected (bottom) AGS cells, NCI-N87 cells and primary gastric epithelial cells. The nuclei were stained with DAPI (blue), scale bar indicate 20 μm. The bar graph below the microscopic images shows quantification of the interaction. (G) As in panel F, gastric biopsy specimens were analyzed using PLA. Biopsy specimens from one uninfected patient (p1) and one H. pylori infected patient (p6) are shown as examples. The bar graph shows show quantification of multiple images of three uninfected (p1–p3) and three H. pylori infected (p4–p6) biopsy specimens obtained from different individuals. Statistical test shows the difference in PLA signal between the patient groups. The nuclei were stained with DAPI (blue), scale bar indicate 20 μm. Levels of significance are indicated as follows: * p<0.05, ** p<0.01 and *** p< 0.001.

To address if APE1 directly regulates Rac1, Rac1 activity was compared in vector control and APE1 suppressed cells. Fig 4A and 4B demonstrate a significant increase in active Rac1 in APE1 suppressed AGS or NCI-N87 cells within 60 min of infection. Overexpression of exogenous APE1 in APE1 suppressed AGS cells resulted in a decrease in Rac1 activity (Fig 4C). To establish whether APE1 binds to Rac1 to inhibit its activity, we immunoprecipitated APE1 and demonstrated that Rac1 interacted with APE1. This interaction was augmented within 30 min of *H*. *pylori* infection (Fig 4D). The enhanced association between APE1 and Rac1 after *H*. *pylori* infection was further confirmed by confocal microscopy showing co-localization of Rac1 and APE1 staining in the cytosol as indicated in the merged image (Fig 4E). Using in situ proximity ligation assay (PLA) we confirmed cytosolic co-localization of APE1 and Rac1 following *H*. *pylori* infection in AGS, NCI-N87 and antral-derived primary gastric cells (Fig 4F). To demonstrate that the findings in cell lines also occur in native human gastric epithelial cells we performed PLA in primary gastric epithelial cells from gastric mucosal biopsy samples (Fig 4G). Our experiments showed that the APE1-Rac1 interaction was greater in biopsy samples from patients infected with *H*. *pylori* compared to those from uninfected control subjects (Fig 4F right panel). Moreover, by performing Co-IP experiments we observed that APE1 interacted with the constitutively active form of Rac1 (V12) but not with the dominant negative form (N17) (S4 Fig). From these observations we conclude that APE1 negatively regulates activation of Rac1.

### APE1 inhibits H. pylori-mediated Nox1 induction

**Fig 5:**
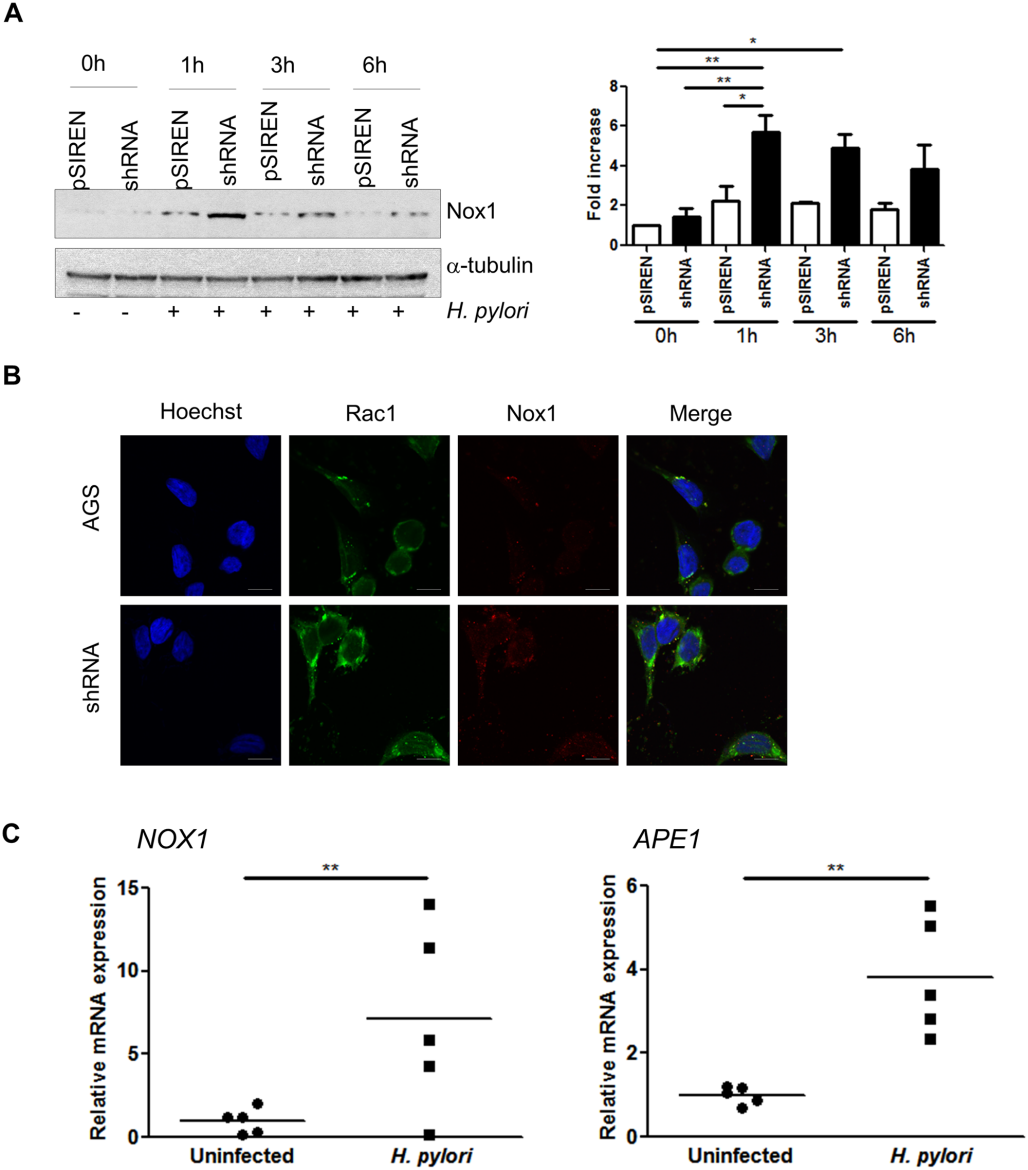
APE1 regulates Nox1 expression. (A) Nox1 expression was examined in AGS pSIREN and shRNA cells infected with H. pylori for 1, 3 and 6h or left uninfected (0h). Corresponding densitometry results of three experiments are shown to the right of the western blot. (B) Immunofluorescence staining showing Rac1 and Nox1 after infection in AGS and shRNA cells. Scale bars indicate 10 μm. (C) Gene transcription levels of Nox1 (left panel) and APE1 (right panel) in gastric biopsies from H. pylori infected and uninfected patients. Each value was normalized to 18S and data were normalized to one of the uninfected samples. Levels of significance are indicated as follows: * p<0.05 and ** p<0.01.

To examine the effect of the level of APE1 on the previously reported increase of Nox1 after *H*. *pylori* infection [13], levels of Nox1 were assessed by western blot in AGS cells with varying APE1 levels after infection at various times. Increased levels of Nox1 were observed in the APE1 suppressed cells compared to the vector control cells within 1h of *H*. *pylori* infection (Fig 5A). This was confirmed by immunofluorescence staining that showed increased Nox1 after infection in APE1 suppressed cells compared to controls (Fig 5B). To determine if the observations found in cell lines could be translated to native human gastric epithelial cells, real time RT-PCR for Nox1 and APE1 were performed with the total RNA isolated from gastric antral biopsies from uninfected or *H*. *pylori* infected patients. The expression of Nox1 and APE1 was significantly increased in tissue from infected patients (Fig 5C). These *in vivo* data suggest a role for Nox1 and APE1 in the response to infection of human stomach with *H*. *pylori*.

### N-terminal lysines of APE1 are crucial to regulate Rac1

**Fig 6:**
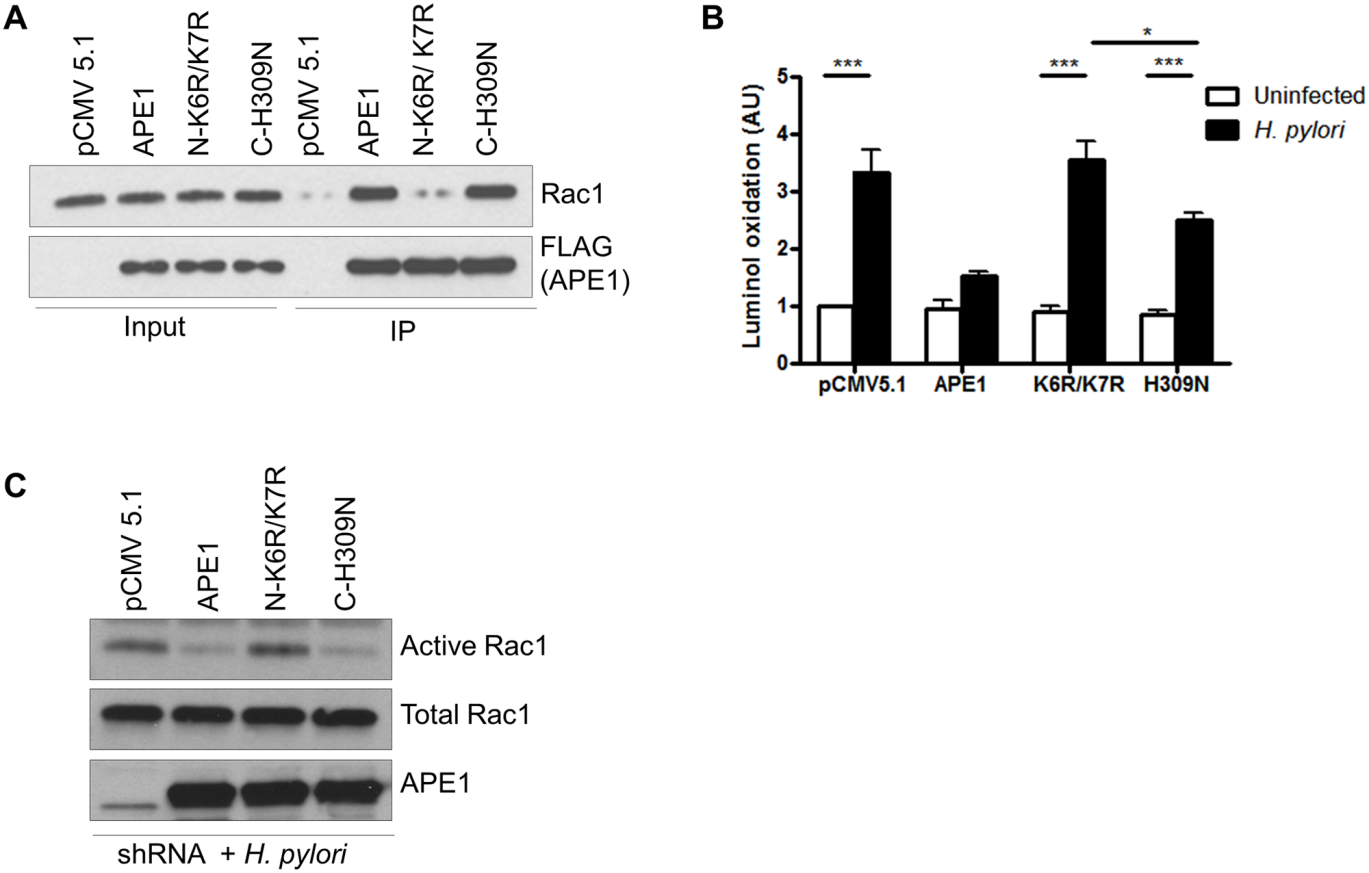
Differential regulation of Rac1 activity and ROS generation by two functional domains of APE1. (A) AGS cells were transfected with either vector (pFLAG-CMV-5.1), WT APE1, or K6R/K7R or H309N mutants and infected with H. pylori for 1h. The cell lysate was immunoprecipitated with anti-FLAG beads and analyzed for Rac1 and FLAG levels by western blot. Part of the cell lysate was used as an input to show the endogenous level of Rac1 and the corresponding APE1-FLAG expression. (B) The same set of transfected shRNA cells from (A) were infected with H. pylori for 30 min or left uninfected and ROS was measured by luminol oxidation. Mean values (± SEM) of five independent experiments are shown. (C) shRNA cells were transfected as in (A) and then infected with H. pylori for 1h and Rac1 activity was measured. A representative western blot was selected from three independent experiments. Levels of significance are indicated as follows: * p<0.05 and *** p< 0.001.

Earlier we established that various regulatory functions of APE1 are largely regulated by its N-terminal lysines (K6K7) and C-terminal histidine (H309) [26]. Therefore, co-immunoprecipitation was performed in AGS cells to determine the binding of Rac1 with the acetylation mutant (N-K6R/K7R) and the DNA repair mutant (C-H309N) of APE1. Our results showed that the N-terminal acetylation mutant of APE1 had minimal binding with Rac1 whereas the binding of the DNA repair mutant was comparable to that of WT APE1 (Fig 6A). To establish if this interaction between APE1 and Rac1 is essential in regulating *H*. *pylori*-induced ROS generation, ROS were measured in APE1 suppressed AGS cells that were transfected with WT APE1, N-terminal mutant or C-terminal mutant and then infected with *H*. *pylori*. We observed a greater than 2-fold ROS increase in the N-terminal mutant overexpressing cells compared to WT APE1. Although overexpression of the C-terminal mutant also showed increased ROS generation compared to WT APE1, this was significantly less than the N-terminal mutant (Fig 6B). To determine if Rac1 activity is modulated by the non-acetylatable mutant of APE1, APE1 suppressed AGS cells were similarly transfected as described in Fig 6B, and Rac1 activation was measured after 30 min of *H*. *pylori* infection. Analogous to the findings of ROS generation, we observed that the non-acetylatable mutant was unable to inhibit Rac1 activation compared to WT APE1 or the DNA repair mutant of APE1 (Fig 6C).

### ROS is generated in APE1 suppressed colonic epithelial cells after microbial infection

**Fig 7:**
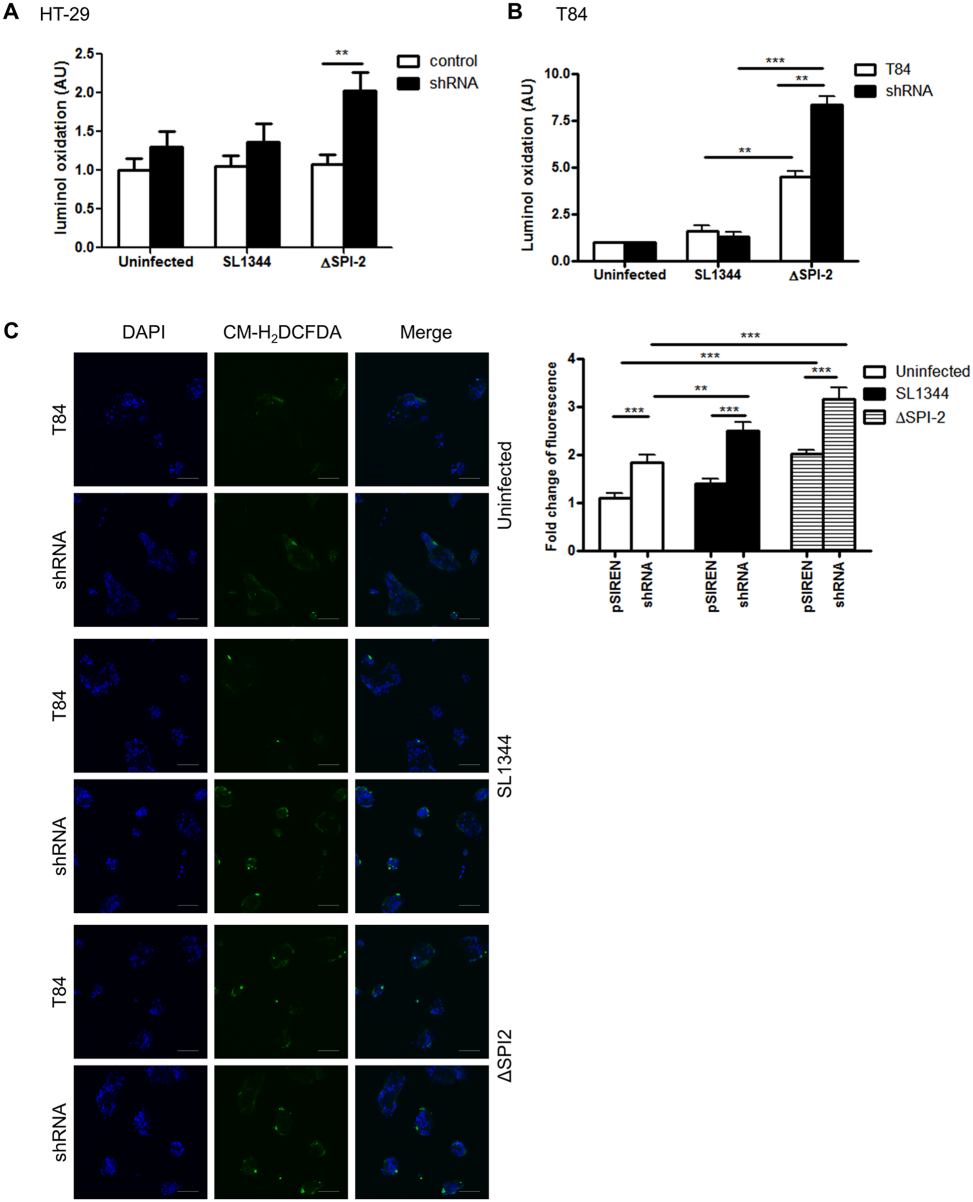
APE1 inhibits ROS generation in gut epithelial cells following infection with Salmonella enterica serovar Typhimurium (SL1344). (A) HT-29 cells and (B) T84 cells were infected with Salmonella SL1344 or ΔSPI2 mutant for 30 min or left uninfected. Fold change of luminol oxidation compared to uninfected cells are shown as mean values (± SEM) of three independent experiments. (C) ROS generation detected by CM-H_2_DCFDA was analyzed by confocal microscopy in WT or shAPE-transfected T84 cells after infection with SL1344 and ΔSPI2 for 30 min or left uninfected. DAPI was used for nuclear staining; scale bar indicates 100 μm. Levels of significance are indicated as follows: ** p<0.01 and *** p< 0.001.

To determine if the suppression of ROS production by APE1 occurs in other epithelial cells within the gastrointestinal tract and with other infections, we generated stable APE1 suppressed human colonic epithelial T84 cells and compared responses to wild type *Salmonella* SL1344 and the *Salmonella* ΔSPI2 mutant. The ΔSPI2 mutant of *Salmonella* was used for the ability of the pathogenicity island 2 of *Salmonella* to inhibit ROS production in phagocytes [34]. For both HT-29 and T84 colonic epithelial cells, we found that infection with ΔSPI2 mutant of *Salmonella* generated ROS that was further increased in corresponding APE1 suppressed cells (Fig 7A and 7B). Compared to the ΔSPI2 mutant, limited amounts of ROS were induced by wild type *Salmonella*. Also, immunofluorescence with CM-H_2_DCFDA demonstrated increased ROS generation in APE1 suppressed T84 cells following ΔSPI2 mutant infection (Fig 7C). This finding suggests that in addition to interfering with Nox2 in macrophages, *Salmonella* may also interfere with the Nox1 complex in intestinal epithelial cells.

## Discussion

In this study we show that APE1 regulates the induction of reactive oxygen species (ROS) by gastroenteric pathogens in a panel of relevant human gastrointestinal epithelial cells. Multifunctional APE1 was demonstrated to inhibit Nox1-mediated ROS production through its direct interactions with Rac1. In addition to preventing formation of the functional NADPH complex, APE1 limits ROS production by decreasing Nox1 expression. Together, these data support the concept that through its molecular interactions with Rac1, APE1 provides negative feedback on Nox1 and oxidative responses in the gastrointestinal epithelium during bacterial infection. These data implicate APE1 in novel molecular interactions that regulate early stress responses elicited by microbial infections.

Microbial pathogens affect host cells through the generation of various radicals [3,35,36]. For example, we and others have demonstrated that *H*. *pylori* infection stimulates the accumulation of intracellular ROS in human gastric epithelial cell lines and freshly isolated native human gastric epithelial cells [37,38]. The potential roles of VacA and CagA in regulating ROS production in cells are also illustrated by other reports showing VacA-dependent regulation of autophagy and associated ROS production [39,40]. In our studies *H*. *pylori* lacking VacA had no significant effect on ROS production as assessed by luminol oxidation. Although CagA has been implicated in increased levels of ROS, the 8–1 mutant lacking CagA did not significantly alter ROS production in our assays [41]. Since dyes that detect ROS species have varying sensitivities and detect ROS in intracellular or extracellular compartments the role of VacA or CagA in the generation of ROS was not conclusively demonstrated in our studies [38,41]. Commensal bacteria that reside in the gut are reported to induce ROS generation from intestinal epithelial cells [42]. High levels of ROS are associated with molecular damage to cellular components and consequent tissue injury but APE1 may represent an important host factor to limit this damage. The differences in the kinetics of ROS generation in the various cell lines employed in this study could be resolved in future studies in animal models. This is particularly relevant to model the persistent infection of humans with *H*. *pylori*.

Advancing our prior observations showing that *H*. *pylori*-induced apoptosis is inhibited by APE1 [26], the present work establishes a novel role of APE1, mediating the inhibition of oxidative stress. This function of APE1 may contribute to its ability to inhibit oxidative stress-induced cell death as well as a fine-tuning of the redox-sensitive responses induced during infection [43]. Although APE1 is referred to as a stress response molecule [44], concordant with a recent report showing the regulation of stress by APE1 in the mitochondria of neuronal cells [45] our work demonstrates its role in regulating stress generation in gastrointestinal epithelial cells.

To understand the mechanism of APE1 as a determinant of ROS regulation, we focused on Rac1 and Nox1, two major contributors of ROS generation in non-phagocytic cells. The small GTPases, Rac1 and Rac2, are common mediators of NADPH-dependent ROS production in diverse signaling pathways that lead to mitogenesis, gene expression and stress responses [18,46]. Our findings corroborate the dependence of Rac1 on ROS production as we show that *H*. *pylori*-induced ROS generation is downregulated by the APE1-Rac1 interaction that subsequently inhibits Nox1. Further characterization of the molecular association between active Rac1, cellular ROS levels and APE1 provides new mechanistic insight into the control of redox-sensitive host responses with potential relevance to the development of novel therapies for gastrointestinal infections and associated inflammation.

As Rac1 is an integral part of the functional NADPH oxidase complex [14], inhibition of Rac1 activity by APE1 is expected to interfere with this assembly, thereby providing negative feedback on ROS generation. Regulation of Rac1 by APE1 was observed in AGS and NCI-N87 cells, however, the kinetics of the regulation of Rac1 and APE1 were different. Although those kinetics varied somewhat, intracellular co-localization of APE1 and Rac1 following infection assessed by using the proximity ligation assay showed a significant increase in both AGS and NCI-N87 at 1h after infection. This co-localization of APE1 and Rac1 was also observed in antrum-derived primary gastric epithelial cells. Overexpression of APE1 decreased ROS comparable with the effect of DPI or of NSC23766 (Fig 3C and 3D), underlining that APE1 is a major regulator of the Rac1-NADPH oxidase axis of ROS production. In addition to Rac1 inhibition, we identified another level of inhibition by APE1 when APE1 suppressed cells were found to express significantly more Nox1 compared to the vector control cells. The observation of an augmentation of *H*. *pylori*-induced ROS generation in two different APE1 suppressed gastric epithelial cells supports a broadly relevant role for APE1 in regulating ROS. Interestingly, APE1 and the phytochemical Ginko biloba both regulated mitochondrial oxidative stress in neuronal cells [45]. APE1 and phytochemical-mediated regulation of mitochondrial oxidative stress could also be of relevance in *Helicobacter-*induced ROS generation in gastric epithelial cells.

Given APE1’s multiple functions, it is not surprising that interacting molecular partners of APE1 have already been identified. It appears likely that acetylation-mediated conformational changes in APE1's N-terminal domain modulate its interaction with partner proteins, including Rac1 [47]. We have not manipulated the various redox-responsive cysteine residues of APE1 in our study. As various reports show a role for the redox function of APE1 in regulating responses to cell stress, the redox function of APE1 may also be involved in cellular responses to oxidative stress. Unlike the stable interaction between APE1 and Rac1, the minimal association between Rac1 and the N-terminal acetylation mutant of APE1 underscores the necessity of the Lys residues for the interaction. Our data indicate that this interaction is essential for the ability of APE1 to inhibit the production of ROS since significantly increased ROS generation was found with the non-interacting acetylation mutant compared to WT APE1 (Fig 6B). Taken together with our previous observation that *H*. *pylori* induced APE1 acetylation [21], this finding highlights a previously unrecognized modification of regulatory molecules during infection. We speculate that the role of APE1 could be similar to the Rho-GDP dissociation inhibitors (Rho-GDI), which translocates Rac1 from the membrane to the cytoplasm, effectively deactivating NADPH oxidase [48,49].

Our observation of the inhibition of Rac1 by APE1 in intestinal cell lines indicates that APE1-regulated ROS generation is conserved between gastric and intestinal epithelial cells. These data suggest a common role for APE1 in the pathogenesis of various prolonged gastrointestinal bacterial infections. Unlike the robust ROS generation typically induced by acute infection, lower levels of ROS produced by host epithelial cells are increasingly recognized to play a critical physiological role [18] including regulation of the molecular machinery of epithelial secretory lineages and autophagy [50]. As such, redox signaling through Nox1 represents a unique intracellular regulator of diverse signaling pathways involved in normal cell physiology, inflammation and carcinogenesis. Due to the nature of *in vitro* infection models, including uncontrolled bacterial growth and related cell stress-induced mitochondrial ROS production in cell models, future experiments *in vivo* are needed to determine the physiological importance of acute versus chronic infections with *H*. *pylori* in relation to regulation of oxidative stress by APE1.

In summary, we have shown that APE1 controls the regulation of epithelial responses to gastroenteric infections and the subsequent generation of oxidative stress. Our findings provide new insights into APE1’s role as a host molecule that modulates ROS generation via negative regulation of Rac1 and Nox1. Our future studies will aim to examine models of prolonged infection and the physiological responses to infections.

## Supporting Information

S1 Fig ROS production following infection with wild type, VacA or CagA mutant *H*. *pylori*. AGS cells were infected with wild type *H*. *pylori* 26695 or *H*. *pylori* lacking VacA or CagA (8–1) for 30 min or left uninfected. ROS was measured by luminol oxidation. Corresponding graphs are shown as the fold change compared to the uninfected cells set to an arbitrary value of 1 (mean ± SEM, n = 3; * = p <0.05).
(TIF)

S2 Fig Prolonged ROS production in AGS cells. AGS cells were infected at an MOI of 100 with *H*. *pylori 26695* or left uninfected. Luminol oxidation was recorded up to 6 h following infection. Graphs are shown as the fold change compared to the uninfected cells set to an arbitrary value of 1 (mean ± SEM, n = 3; * = p <0.05).
(TIF)

S3 Fig Activation of Rac1 by wild type or CagA mutant *H*. *pylori*. AGS cells were infected with *H*. *pylori* 26695 and *H*. *pylori* 8–1 for 30 min or left uninfected and Rac1 activity was measured. Representative immunoblot showing active Rac1 and total Rac1 levels.
(TIF)

S4 Fig Immunoprecipitation of APE1 with V12-FLAG or N17-FLAG with or without infection. AGS cells were transfected with pcDNA or V12-FLAG or N17-FLAG and then infected with *H*. *pylori* for 1 h before immunoprecipitation with the anti-FLAG M2 agarose beads. Representative western blot is showing the endogenous APE1 level and the corresponding FLAG expression.
(TIF)

S1 Supplementary Materials and Methods Additional information is provided for the following procedures: Measurement of ROS, Western blotting and immunoprecipitation, Measurement of Rac1 activation, Real time RT-PCR from gastric biopsies, Confocal Microscopy, Proximity ligation assay (PLA) by confocal microscopy. (DOCX)
