# Supplementary figures and images for: Regulation of Rac1 and Reactive Oxygen Species Production in Response to Infection of Gastrointestinal Epithelia
Source: PLoS Pathog. 2016 Jan 13;12(1):e1005382. doi: 10.1371/journal.ppat.1005382 (PMC4711900; doi:10.1371/journal.ppat.1005382)

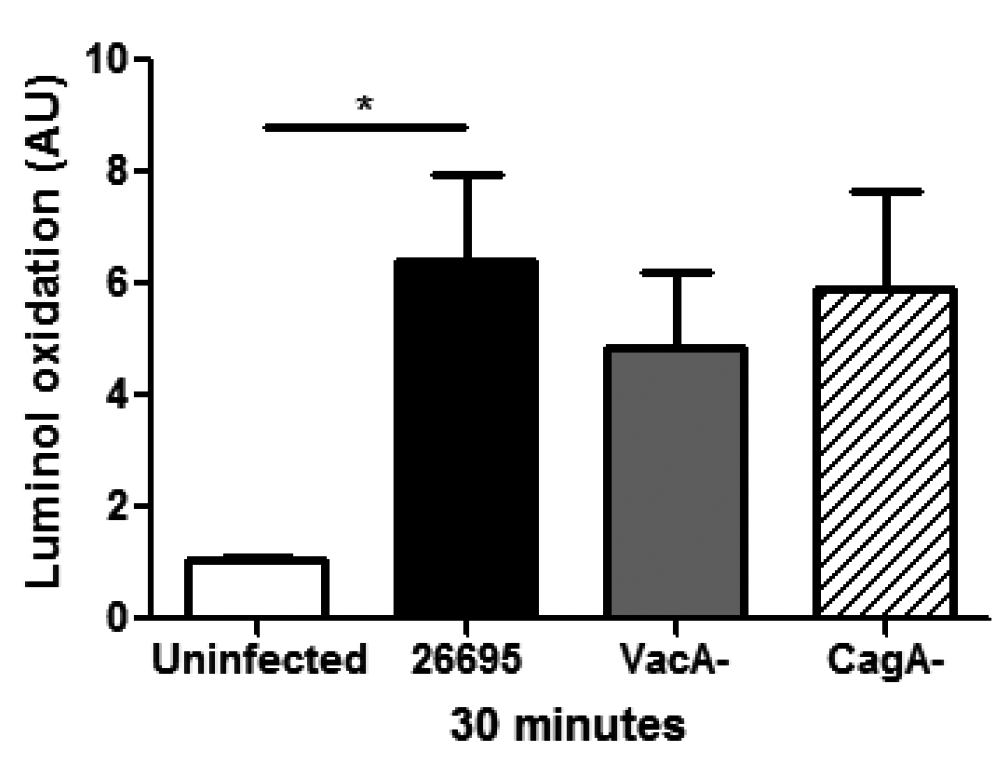

Supplement: S1 Fig — AGS cells were infected with wild type H. pylori 26695 or H. pylori lacking VacA or CagA (8–1) for 30 min or left uninfected. ROS was measured by luminol oxidation. Corresponding graphs are shown as the fold change compared to the uninfected cells set to an arbitrary value of 1 (mean ± SEM, n = 3; * = p <0.05). (TIF) [file ppat.1005382.s001.tif]

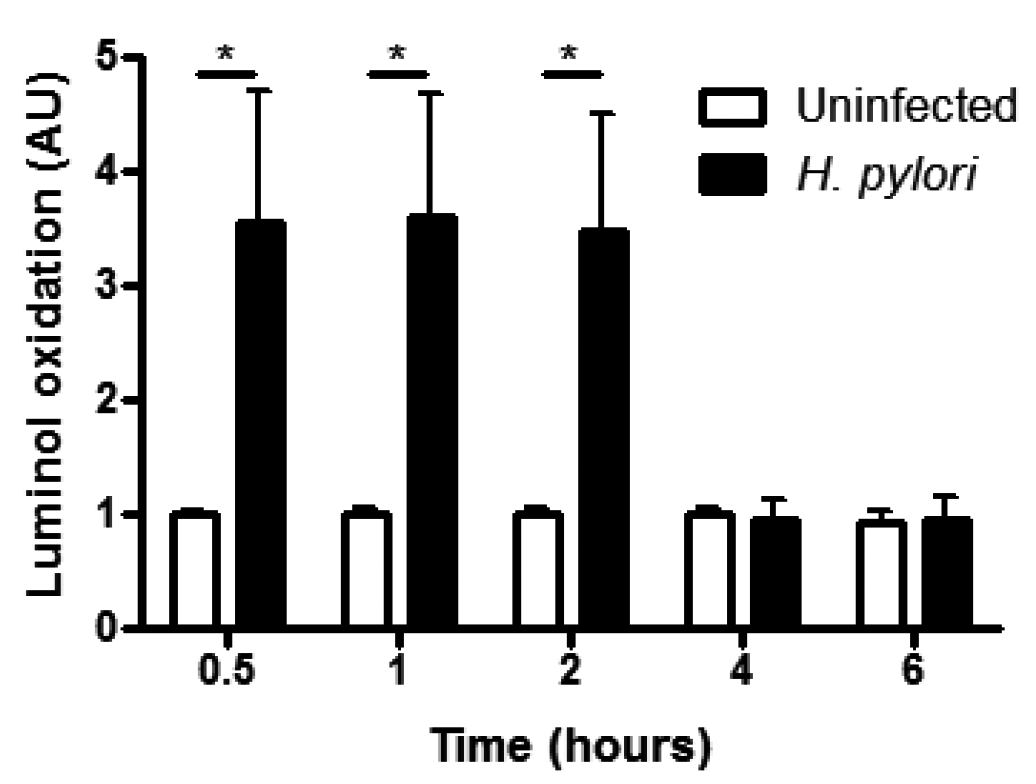

Supplement: S2 Fig — AGS cells were infected at an MOI of 100 with H. pylori 26695 or left uninfected. Luminol oxidation was recorded up to 6 h following infection. Graphs are shown as the fold change compared to the uninfected cells set to an arbitrary value of 1 (mean ± SEM, n = 3; * = p <0.05). (TIF) [file ppat.1005382.s002.tif]

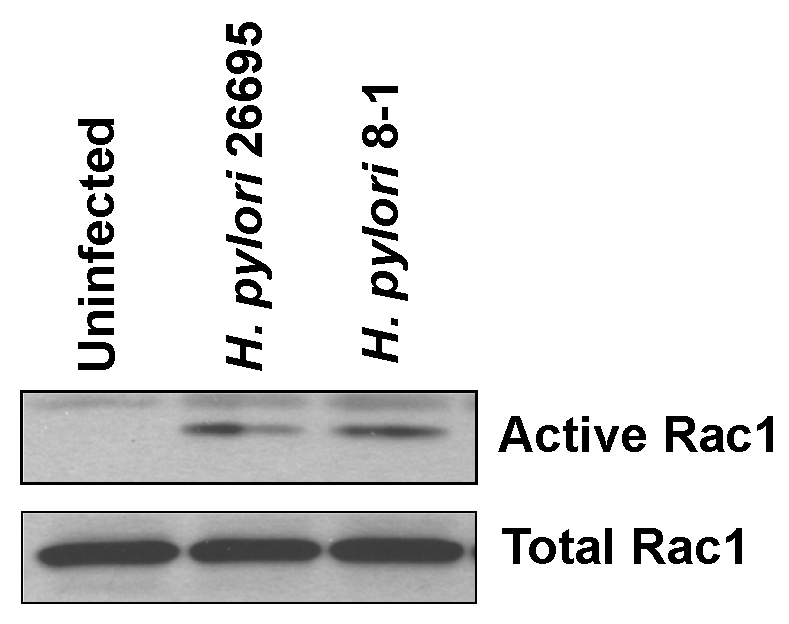

Supplement: S3 Fig — AGS cells were infected with H. pylori 26695 and H. pylori 8–1 for 30 min or left uninfected and Rac1 activity was measured. Representative immunoblot showing active Rac1 and total Rac1 levels. (TIF) [file ppat.1005382.s003.tif]

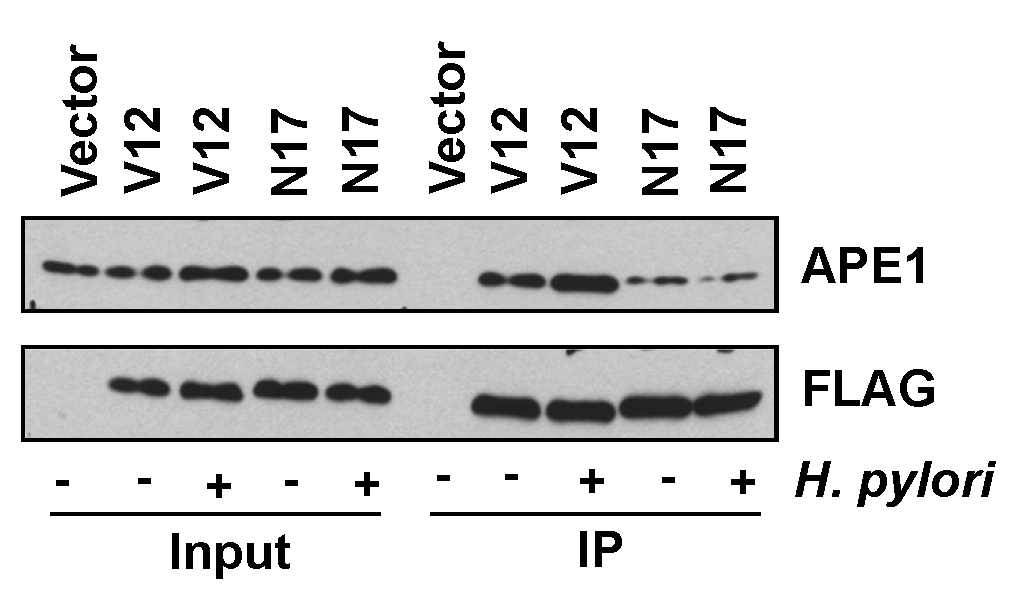

Supplement: S4 Fig — AGS cells were transfected with pcDNA or V12-FLAG or N17-FLAG and then infected with H. pylori for 1 h before immunoprecipitation with the anti-FLAG M2 agarose beads. Representative western blot is showing the endogenous APE1 level and the corresponding FLAG expression. (TIF) [file ppat.1005382.s004.tif]
