# Supplementary material for: Regulation of Rac1 and Reactive Oxygen Species Production in Response to Infection of Gastrointestinal Epithelia
Source: PLoS Pathog. 2016 Jan 13;12(1):e1005382. doi: 10.1371/journal.ppat.1005382 (PMC4711900; doi:10.1371/journal.ppat.1005382)
Supplement: S1 Supplementary Materials and Methods — (DOCX) [file ppat.1005382.s005.docx]

**Supplementary Materials and Methods**

**Measurement of ROS**

Intracellular ROS was measured according to the protocol described in Lumimax Superoxide Anion Detection Kit (Stratagene). Briefly, 5x 10^5^ cells were infected with *H. pylori* with or without DPI (10µM, 1h pre-treatment) , N-acetylcysteine (NAC, 10 mM, 1h pre-treatment), NSC23766 (100 µM, overnight pre-treatment) or left uninfected. Subsequently, the cells were suspended in 100 µl of supplied assay media and 100 µl combined luminol and enhancer solution was added as per the protocol. Total cell suspension was incubated for 10 min and the luminescence was measured using a standard luminometer (Monolight 2010, Analytical Luminescence Laboratory).

**Western blotting and immunoprecipitation**

Proteins were fractionated on SDS-PAGE, transferred onto nitrocellulose membrane (Bio-Rad), blocked with 5% non-fat dry milk in TBST buffer (20 mM Tris base, 150 mM NaCl, 0.05% Tween 20, pH adjusted to 7.6 with HCl) for 1 h and probed with primary antibody followed by incubation with anti-rabbit or anti-mouse HRP-conjugated IgG (Cell Signaling Technology) at 1:2000 dilution. Immunoreactions were visualized by chemiluminescence (Cell Signaling Technology). Protein loading was normalized to α-tubulin using its antibody (Abcam).

For immunoprecipitation, 1 × 10^6^ cells were plated in 6 well plates. Following transfection, treatments or infections, cells were washed with PBS, lysed with 250 µl RIPA buffer (150 mM NaCl, 50 mM Tris Cl- pH 7.4, 1% NP-40, 0.1% sodium deoxycholate, 1 mM EDTA with protease inhibitor cocktail) by keeping on ice for 30 min. Lysates were clarified at 13,000 × g for 10 min at 4ºC. 500 µg of cell lysates were incubated with 30 µl anti-FLAG M2 agarose beads (sigma) for 4h at 4ºC. The agarose-bound immunocomplex was washed three times with the same lysis buffer, boiled and resolved by SDS-PAGE, followed by western blot to detect associated proteins.

**Measurement of Rac1 activation**

Rac1 activity assay was performed as described previously ([31](#_ENREF_31)). Briefly, infected or uninfected cells were lysed using 50 mM Tris-HCl (pH 7.5), 2 mM MgCl_2_, 0.1 M NaCl, 1% NP-40, and 10% glycerol with protease inhibitors buffer and incubated with Glutathione S-transferase (GST) coupled to the p21-binding domain of Pak (PBD) to precipitate Rac-GTP. Beads were then washed 4 times in lysis buffer and resuspended in SDS sample buffer. Samples were then loaded onto SDS-PAGE and processed for Western blotting.

**Real time RT-PCR from gastric biopsies**

Total RNA was extracted using the RNeasy kit (Qiagen, Valencia, CA) and reverse transcribed using the Superscript kit (Invitrogen), both according to the manufacturers’ instructions. Real-time probe PCR for human Nox1 and APE1 was performed in a SmartCycler (Cepheid, Sunnyvale, CA) using primers and FAM-labeled probe sets purchased from Applied Biosystems. Following amplification, mRNA expression levels were determined semi-quantitatively by comparing the critical threshold (CT) values to a standard curve and normalizing these CT values against 18S rRNA CT values.

**Confocal Microscopy**

AGS cells were seeded at a density of 2.0X10^5^ cells/ml on sterile 18 mm square # 1.5 cover slips in six-well plates. The following day, cells were exposed to *H. pylori* strain 26695 for 1 hr at a MOI 300. Samples were washed in 1X PBS, pH 7.4 and fixed in 2% formaldehyde, washed with PBS, and permeabilized with 0.1% triton in PBS for 5 min. Cells were blocked with 5% goat serum -1.5% BSA in PBS (blocking solution) and subsequently incubated with mouse-anti-Rac1 (Millipore clone 23A8) diluted 1:200 and rabbit-anti- APE1 antibody (Cell Signaling) diluted 1:100 in in blocking solution overnight at 4°C. Cells were washed 4 times with 0.0005% triton® X-100 in PBS and incubated with Alexa Fluor® 488 goat anti-mouse IgG H and L and Alexa Fluor® 555 goat anti-rabbit IgG H and L, highly cross-adsorbed, 2 mg/mL (Life Technologies) both diluted 1:1000 in blocking solution for 1hr. Cells were washed 5 times with 0.0005% triton® X-100 in PBS. Cells were incubated for 5 min in Hoechst 33342 (Life Technologies) diluted 1:2000 in 1X PBS to stain the nuclei. Cells were washed with 1X PBS and subsequently mounted on glass slides with prolong gold (Life Technologies). Confocal images were obtained with a 100X objective and a 2.5X zoom using an Olympus FV1000 Confocal microscope system (UCSD neuroscience, grant # NS047101) controlled by FV10-ASW 3.0 FV1000 software (Olympus). Hoechst staining was visualized with a 405 nm laser, Rac1 was visualized with a 488 nm laser, and APE1 with a 543 nm laser. After image acquisition, single plane, merged and z-stacks were processed with FV1000 software (Olympus).

**Proximity ligation assay (PLA) by confocal microscopy**

APE1-Rac1 interactions were detected with Duolink PLA Kit (Olink Bioscience, Uppsala, Sweden: PLA probe anti-rabbit plus; PLA probe anti-mouse minus; Detection Kit orange) according to the manufacturer's protocol. Briefly, AGS cells were grown, fixed and permeabilized as described for confocal microscopy. The samples were incubated with primary rabbit-anti-APE1 polyclonal antibody (1:200) and mouse-anti- Rac1 monoclonal antibody (1:100) as in confocal microscopy for 2h at room temperature. After the last washing step, the samples were mounted using supplied mounting media containing DAPI. Confocal microscopy was performed and analyzed as described in the previous section.
